# Long-Term Outcomes of Ultrasound-Guided Hydrodistension for Adhesive Capsulitis: A Prospective Observational Study

**DOI:** 10.3390/tomography9050147

**Published:** 2023-10-14

**Authors:** Sofia Dimitri-Pinheiro, Michail E. Klontzas, Evangelia E. Vassalou, Madalena Pimenta, Raquel Soares, Apostolos H. Karantanas

**Affiliations:** 1 Radiology Department, Portuguese Institute of Oncology of Porto—Francisco Gentil EPE, Rua Dr. António Bernardino de Almeida, 4200-072 Porto, Portugal; dimitrisofia@hotmail.com; 2 Unit of Biochemistry, Biomedicine Department, Faculty of Medicine, University of Porto, Alameda Professor Hernâni Monteiro, 4200-319 Porto, Portugal; raqsoa@med.up.pt; 3 Department of Medical Imaging, University Hospital of Heraklion, 71110 Heraklion, Crete, Greece; miklontzas@gmail.com (M.E.K.); vassalou.e@hotmail.com (E.E.V.); 4 Department of Radiology, School of Medicine, University of Crete, Voutes Campus, 71003 Heraklion, Crete, Greece; 5 Radiology Department, São João Hospital Centre, Alameda Prof. Hernâni Monteiro, 4200-319 Porto, Portugal; madalenapimenta@yahoo.com; 6 I3S—Institute for Innovation and Health Research, University of Porto, Rua Alfredo Allen 208, 4200-135 Porto, Portugal

**Keywords:** adhesive capsulitis, frozen shoulder, ultrasound-guided treatment, hydrodistention, diabetes mellitus, recurrence, follow-up

## Abstract

Ultrasound-guided hydrodistention has been established as an effective minimally invasive treatment option for glenohumeral joint adhesive capsulitis (AC). Nonetheless, the long-term outcomes of the procedure have not yet been established. A total of 202 patients with AC were prospectively recruited and followed up for a total of 2 years. Pain and functionality were assessed with the use of the visual analogue scale (VAS) and the disabilities of the arm, shoulder, and hand (DASH) score, respectively, at the beginning and the end of the follow-up period. The relapse of AC over the 2-year period and the effect of diabetes were also evaluated in the treatment cohort. The Mann–Whitney U test was used to compare mean scores at the two time points, and Cox survival analysis and χ^2^ test were used to assess the effect of diabetes on AC relapse. VAS and DASH scores were significantly lower at 2 years compared with the beginning of the follow-up period (*p* < 0.001). Diabetes was diagnosed in 38/202 patients (18.8%) and was found to be significantly associated with recurrence of the disease (*p* < 0.001). In conclusion, in this observational study, we have demonstrated that ultrasound-guided hydrodistention is linked to excellent long-term outcomes for the treatment of AC, which are significantly worse in patients with diabetes.

## 1. Introduction

Adhesive capsulitis (AC) of the glenohumeral joint is commonly known as “frozen shoulder” and affects a significant population segment with an incidence of up to 5% in the general population. AC of the glenohumeral joint has been recognized as a significant cause of shoulder pain and functional impairment for a large number of individuals [1,2]. Delving into the histopathology of AC shows that the disease involves inflamed glenohumeral joint and subacromial synovium, hypertrophy of the coracohumeral ligament, and fibrosis of the joint capsule. However, despite the clear histopathological findings, its pathoetiology is complex with both genetic and environmental factors, which collaboratively contribute to the phenotype of AC playing an important role in the progression of the disease [3]. Despite the fact that AC is, in most cases, a self-limited condition that subsides within a period ranging between 12 and 18 months, a large proportion of patients may suffer from the disease for a period that can reach 7 years from the time of onset [3].

AC is characterized by four distinctive stages, which have been established by a combination of clinical, arthroscopic, and histological data. Each stage has a characteristic clinical and histological appearance. The first stage, the “preadhesive” phase, is encountered up to 3 months from the onset of symptoms. At this early introductory stage, synovitis and hypervascularity of the rotator cuff interval can be found, but the symptoms can be confused with other diseases, especially if a history of trauma coexists. Pain can be described as mild, which is exacerbated with shoulder movements, pronounced during the night and resting states [4,5]. Analgesic and anti-inflammatory medication has been shown to have minimal effect in reducing the symptoms, and the range of motion has not been yet affected. The second stage, the “freezing” phase, is usually encountered between 3 and 9 months after the onset of symptoms. This phase is characterized by the increase in pain compared with stage 1, and histology demonstrates synovial tissue proliferation. This marked increase in pain indicates that the patient has entered stage 2 [4]. The hallmark of the third stage, the “frozen” phase, is the significant reduction in range of motion. At this stage, adhesions have formed and matured, restricting the motion of the glenohumeral joint [4,5]. Even though that synovitis of the previous stages has significantly subsided, pain is still present but potentially more limited compared with the first and second stages. This third stage is usually encountered between 4 months and 1 year after the onset of symptoms. Finally, the fourth stage, known as the “thawing” phase, can last for 3 or more years after the end of phase 3 and is characterized by marked retraction of the capsule of the glenohumeral joint. Pain has subsided in most of the cases, and there is steady improvement in range of motion, which may never recover to the pre-AC level. Understanding and being able to recognize these stages is important in diagnosing and managing AC, leading to better outcomes for the patient [4,5].

The disease typically affects female patients in their fourth–sixth decade of life and can be classified into primary (idiopathic) or secondary. Secondary adhesive capsulitis (SAC) has been extensively studied and is known to be associated with a variety of underlying predisposing factors. Some of the primary culprits linked to the onset of SAC include extended periods of immobilization, which can be especially prevalent in patients who have undergone prolonged bed rest or limited movement. In addition, a history of surgical interventions or any form of trauma directly affecting the shoulder joint has been noted to contribute to the development of this condition. Furthermore, rotator cuff pathologies, which encompass a range of shoulder disorders affecting the muscles and tendons that stabilize the joint, are recognized as significant risk factors. There is also evidence pointing to thyroid dysfunction as a possible trigger. On a broader scale, both cerebrovascular diseases, which pertain to conditions impacting blood vessels supplying the brain, and systemic inflammatory diseases, which involve inflammation throughout the body, have been associated with SAC [4]. Moreover, individuals diagnosed with diabetes mellitus have been found to have an increased susceptibility to this ailment, among various other contributing factors [4,5]. It is intriguing to note that patients diagnosed with both type I and type II diabetes mellitus exhibit a notably higher predisposition to AC. Specifically, their likelihood of developing AC is magnified roughly fivefold when contrasted with those who do not suffer from diabetes. Furthermore, a deeper delve into the clinical experiences of these diabetic patients reveals that they not only manifest more intense and debilitating symptoms but also endure a more extended and arduous progression of the disease. Moreover, when their treatment outcomes are juxtaposed with those of nondiabetic individuals, it becomes evident that the therapeutic results in diabetic patients are often less favorable. This underscores the significant impact of diabetes on the severity and management of AC [6,7,8].

The diagnosis of AC is mainly clinical with the identification of pain for at least 4 weeks and reduced range of motion in at least three planes. Pain related to AC can be localized at various locations around the shoulder and is typically present during the night and at rest [8]. Nonetheless, despite the fact that imaging is not crucial for the diagnosis, both ultrasound and MR imaging can be used to exclude other conditions that may mimic AC, such as arthropathy of the acromioclavicular joint, glenohumeral arthropathy of degenerative or inflammatory origin, rotator cuff pathology including hydroxyapatite deposition disease, subacromial–subdeltoid bursitis, and cervical spinal pathology. In addition, signs of AC can be recognized on both MR imaging and US [9,10,11]. MR imaging can indicate a thickened coracohumeral ligament, thickening of the axillary recess with surrounding soft-tissue edema, and obliteration of the normal fat of the rotator cuff interval where increased signal on T2-w or PD-w fat-suppressed images can be demonstrated. Abnormal enhancement following contrast medium administration is constantly seen in the rotator cuff interval and the axillary recess. On the other hand, US can be used both to exclude other types of pathology mimicking AC and to apply US-guided treatments. Findings consistent with the diagnosis of AC on US include a thickened coracohumeral ligament, thickening of the joint capsule and reduced capacity of the axillary recess, increased Doppler signal in the rotator cuff interval, and reduction of normal infraspinatus tendon sliding on dynamic examination. The Doppler signal is not constantly seen, even by experienced musculoskeletal radiologists. Nonetheless, none of these findings are diagnostic of AC, and clinical evaluation is the basis for the diagnosis of the disease. After confirming the diagnosis, US can be used to guide treatment with hydrodistention of the glenohumeral joint being the most important treatment option for the management of these patients [6,7].

Ultrasound is the modality of choice for the guidance of interventional shoulder procedures. Its high resolution, low cost, lack of ionizing radiation, and the ability for dynamic real-time assessment of the patient render ultrasound the first-line modality for shoulder interventional procedures [12]. Hydrodistention of the glenohumeral joint has gained traction and has been put forward as a potential first-line course of action for the treatment for AC, resulting in pain and disability improvement. The major short- and midterm benefits of US-guided hydrodistention have been highlighted in many studies [13,14]. As stated in the recent guidelines by the European Society of Musculoskeletal Radiology (ESSR), US-guided hydrodistention for adhesive capsulitis yields better outcomes than palpation-guided or sham injections for the treatment of adhesive capsulitis [15]. Nonetheless, to the best of our knowledge, there is a significant literature gap with regard to the examination of the long-term efficacy of the method. In fact, a comprehensive literature review identified that only one study to date has taken the initiative to evaluate the efficacy of the method in the long term, reporting on the effectiveness and impact on patients [13]. Additionally, no data exist on the recurrence or relapse rate of AC following an initial hydrodistention procedure. This is vital for a holistic understanding of the procedure’s sustainability and long-term benefits.

The aim of this study was to evaluate a large cohort of patients who have been treated with US-guided hydrodistention for glenohumeral joint AC for a 2-year period. Specifically, we aimed to assess the long-term outcome of US-guided hydrodistension of the glenohumeral joint in terms of pain and function at 2 years’ follow-up and define the rate of AC relapse and the time to recurrence in nondiabetic and diabetic patients with AC.

## 2. Materials and Methods

### 2.1. Patients

Within a 2-year period, a total of 202 patients with AC were prospectively recruited and followed up. AC was clinically diagnosed in cases where shoulder pain was present for at least 1 month with inability to lie on the affected side and reduced range of motion of the affected joint in passive and active movements in at least three planes [15]. Exclusion criteria comprised patients with rotator cuff tears, labral tears, long head of the biceps partial or complete tears, osteoarthritis of the glenohumeral and acromioclavicular joint, tumors, and rheumatological disease of the shoulder and those lost during follow-up or noncomplying with the immediate postprocedural exercise program. All shoulders underwent US examination prior to the interventional procedure to exclude other conditions presenting with reduced range of motion and pain [16]. Patients who were determined to be noneligible were carefully identified using a comprehensive approach. This approach consisted of in-depth clinical evaluations, detailed diagnostic US scans, and, in certain specific instances, the use of MR imaging to corroborate findings. It is noteworthy to mention that within the pool of patients analyzed in this study, there existed a particular subset. A subset of patients examined in this study had been previously assessed for different timeframes and outcomes [6,12]. The research was conducted in line with the Helsinki Declaration and received approval from the Ethical Committee of our University Hospital (18092021). Before inclusion, every patient provided their signed informed consent.

### 2.2. Measurements

Demographic parameters, such as age and sex, were recorded together with the presence of diabetes. The diagnosis of diabetes was determined using a stringent set of criteria to ensure accuracy and reliability. A patient would be diagnosed as diabetic if they met one of the following thresholds:

(1) They exhibited a blood glucose level that equaled or exceeded 126 mg/dL.
(2) The HbA1c test, which provides a long-term view of average blood sugar levels, was another diagnostic tool. If a patient had a measurement of HbA1c that was equal to or surpassed 6.5%, and this reading was observed consistently in two distinct tests, they were diagnosed as diabetic.
(3) An oral glucose tolerance test was also utilized as a diagnostic measure. In this test, patients are administered a glucose solution orally, and their blood glucose levels are subsequently measured. A diagnosis of diabetes was made if, 2 h after the glucose intake, and the patient’s blood glucose levels reached or exceeded 200 mg/dL.

Recurrent AC after initial treatment was defined as either (i) failure to improve or (ii) transient improvement of variable degree followed by symptoms relapse, warranting further medical attention. Failure to improve and symptoms relapse were based on specific criteria as defined for the initial diagnosis of the disease (shoulder pain for at least 1 month, inability to lie on the affected side, reduced range of motion of the affected joint in passive and active movements in at least three planes) rather than on subjective patients’ judgment. The number of cases with AC recurrence and the time-to-recurrence were recorded in both nondiabetic and diabetic cohorts. Visual analogue scale (VAS) score and disabilities of the arm, shoulder, and hand (DASH) score were recorded at initial presentation and 2 years after initial treatment. The online tool at https://orthotoolkit.com/dash/ (accessed on 4 October 2023) was used for the calculation of DASH. The number of hydrodistention repeats in nondiabetic and diabetic patients with AC recurrence was recorded.

### 2.3. Diagnostic US Examination and US-Guided Hydrodistention

Prior to the procedure, all patients were assessed with diagnostic US examination to exclude other diseases causing shoulder pain and reduced range of motion. A GE Logiq E10 and a Siemens ACUSON Sequoia ultrasound system with high-frequency linear array probes were used for patient assessment. Two radiologists performed all diagnostic examinations and procedures (4 and 14 years of experience in MSK ultrasound/intervention) according to the European Society of Musculoskeletal Radiology (ESSR) guidelines [17].

**Figure 1:**
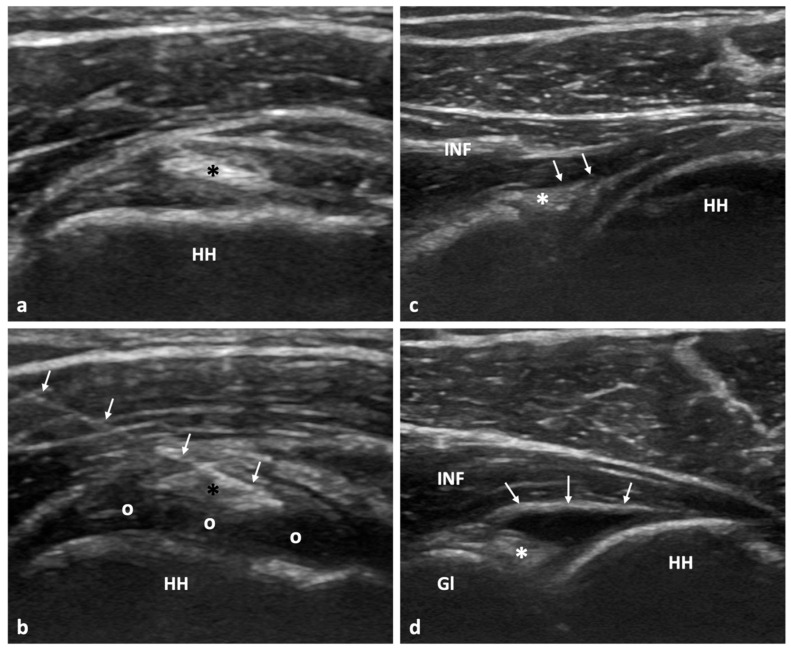
A 42-year-old female patient with clinically diagnosed adhesive capsulitis of the glenohumeral joint. (**a**) Ultrasonographic image along the short axis of the intra-articular portion of the long head of the biceps tendon (*) within the rotator cuff interval. (**b**) Same ultrasonographic plane as in (**a**) showing the proper needle placement (arrows) immediately superior to the long head of the biceps tendon (*). Note the intra-articular accumulation of the anechoic injected solution (o). (**c**) Ultrasonographic image along the long axis of the infraspinatus muscle (INF) showing the posterior lablum (*) and posterior joint recess (arrows) prior to injection. (**d**) Same ultrasonographic plane as in (**c**) showing the distended posterior joint recess (arrows) following the intra-articular injection. The posterior lablum is also clearly evident (*). HH, humeral head; Gl, glenoid.

The US-guided hydrodistention procedure was executed in line with the methods previously outlined in reference [6]. Employing a rigorous aseptic approach that emphasizes a no-touch methodology, the intervention involved precise injection using a 21 G needle. This injection comprised a specific blend of 30–50 mL solution containing 0.9% normal saline (NS), 1% lidocaine, 0.25% bupivacaine, and 40 mg of triamcinolone. The primary objective of this mixture was to ensure that the joint experienced adequate distension without causing any rupture to the capsule, as indicated in reference [13] since capsule rupture has been linked to worse treatment outcomes (Figure 1). After the procedure, a crucial observation period was maintained, during which all patients were closely monitored for a duration of 30 min. This was to ensure that they did not display any immediate adverse reactions. Subsequently, these patients were introduced into an immediate postprocedural physiotherapy session, which aimed to kickstart their recovery process. As previously found [15], immediate postprocedural manipulation is superior to late long-term physiotherapy, which is not the case in other conditions such as calcific tendinopathy [15]. Postprocedural manipulations included a total of 30 min of exercises: (1) a 10 min cycle of alternating passive internal and external rotation and flexion attempting to achieve the maximum range of motion, followed by 10 min of active movements of the patient in a standing position against the wall, alternating between flexion and external rotation. For the final 10 min, the patient continued actively exercising in the room under supervision [15]. Additionally, detailed guidance was provided to each patient. They were advised to apply cryotherapy, which involves cold treatment, for intervals of 20 min. Furthermore, they were instructed to take 1000 mg of paracetamol at regular intervals, specifically every 8 h, and to continue this regimen for a period of 2–3 days to manage pain and inflammation. Apart from hydrodistention and painkillers, no other treatment was offered to our patients for the duration of the 2-year follow-up.

### 2.4. Statistical Analysis

Our study utilized descriptive statistics for the analysis of demographic data, which included age and sex. VAS and DASH scores were recorded at initial presentation and at 2 years of follow-up and were presented as mean ± SD to provide a comprehensive view of the data spread. Comparisons between means and identification of statistical differences were performed with the use of nonparametric Mann–Whitney U tests. Additionally, to delve deeper into the patterns of AC recurrence over the 2-year span, we employed the Cox survival analysis. This analysis was pivotal in discerning the variances between diabetic and nondiabetic patients in terms of AC recurrence. Further, to determine the potential impact of diabetes on the relapse rate of AC, we utilized the Chi-square test. This statistical test is used to study the relationships between categorical variables. All of our data computations and statistical tests were executed using the SPSS software, version 29 for MacOS. Significance was denoted with a *p*-value of less than a = 0.05.

## 3. Results

In this study, 202 patients were prospectively included. This group included a total of 149 female and 53 male patients, with a mean age of 47.3 ± 17.85 years. Assessing the presence of diabetes in our patient group indicated that diabetes was diagnosed in 39/202 patients (19.3%), while recurrence of AC was recorded in 28/202 patients (13.86%) over the 2-year period.

**Figure 2:**
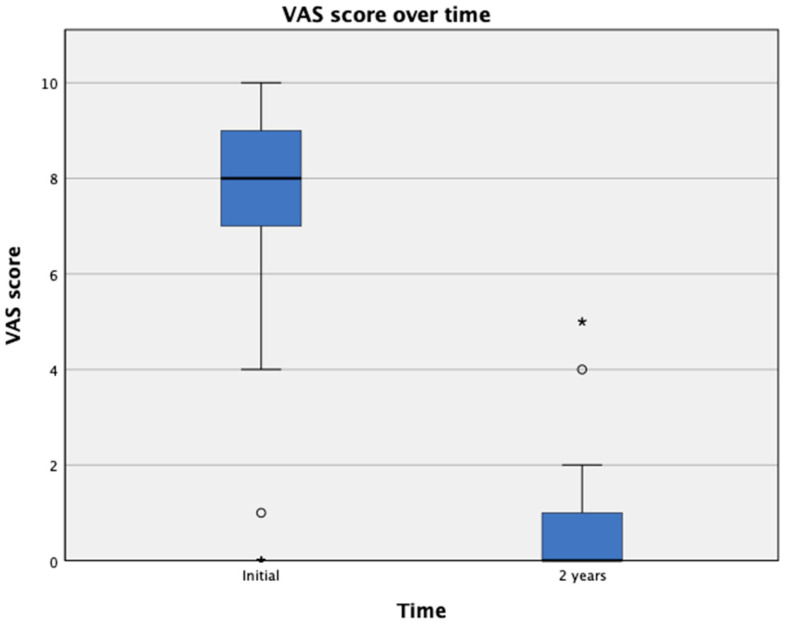
VAS score over time. VAS, visual analogue scale. *: *p* < 0.05.

**Figure 3:**
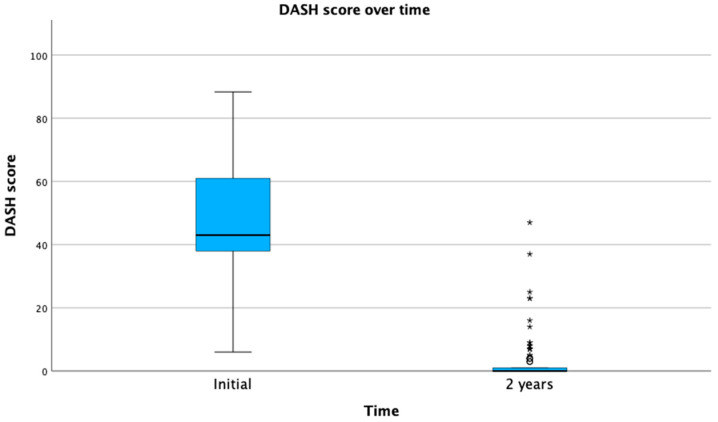
DASH score over time. DASH, disabilities of the arm, shoulder, and hand. *: *p* < 0.05.

**Figure 4:**
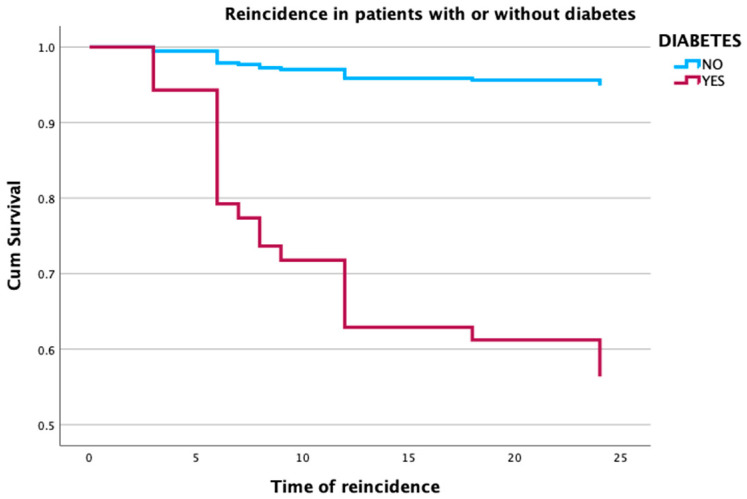
Survival analysis of the time to recurrence for diabetic (red line) and nondiabetic (blue line) patients.

The mean VAS score at presentation was 7.97 ± 1.79 compared with a mean of 0.4 ± 0.7 at 2 years. The mean DASH score was 47.42 ± 18.18 and 1.6 ± 5.4 at initial presentation and 2 years, respectively (Figure 2 and Figure 3). The effect of Cox regression survival analysis showed that patients with diabetes had a significantly lower time to recurrence compared with patients without diabetes (*p* < 0.001) (Figure 4). Diabetes was also significantly associated a higher number of cases presenting with AC recurrence (*p* < 0.001) (Table 1). No immediate or late side effects of the treatment were found in our cohort of patients.

## 4. Discussion

Here, a cohort of nondiabetic and diabetic patients with AC was treated with US-guided hydrodistension and followed up for 2 years. Hydrodistention was linked to excellent long-term outcomes with regard to shoulder pain and functionality. Diabetes was found to be a significant determinant of the treatment outcome, being significantly associated with a higher rate of AC recurrence and lower time-to-recurrence interval compared with nondiabetic patients. To our knowledge, this is the first study supporting the long-term beneficial effect of hydrodistention in a cohort consisting of both nondiabetic and diabetic patients with AC.

Treatment strategies for AC vary widely, and an evidence-based treatment algorithm remains to be defined. A series of treatment options have been proposed over the years for the management of the disease. These include conservative treatment, with oral steroids, nonsteroidal anti-inflammatory drugs, or paracetamol, which aims to reduce inflammation and pain [18]. In addition, physiotherapy alone or in combination with drug therapy has been proposed as an option to restore range of motion in the affected joint [19]. However, neither drug therapy nor physiotherapy has been linked to favorable outcomes. Pharmacotherapy, in particular, can have important side effects related to the use of steroids and anti-inflammatory medication. Surgery has been also traditionally applied for the treatment of AC with arthroscopic release of adhesions or manipulation under general anesthesia. These procedures carry the risk of anesthesia, a long recovery time, and risks related to axillary nerve and articular cartilage and labral damage [2,4,20,21]. These limitations, combined with the fact that their efficacy has not be proven to be higher than other minimally invasive alternatives, have increased the interest for US-guided treatments, including hydrodistention of the joint capsule [21,22].

Among other approaches and especially in the context of failed conservative treatment, hydrodistention of the glenohumeral joint has been shown to be effective in reducing pain and restoring shoulder range of motion [23,24,25]. Although most studies have been focused on the effectiveness of the procedure in the short and midterm, to the best of our knowledge, data regarding the long-term outcomes are sparse. In this context, there is a single reported study by Watson et al. evaluating the efficacy of the method at 2-year follow-up [10]. In this study, the authors assessed the outcome of glenohumeral joint hydrodistention in 41 patients with AC, excluding those with diabetes mellitus, at baseline, 3 days, 1 week, 3 months, 1 year, and 2 years after treatment. Injected solution consisted of a mixture of steroid (40 mg triamcinolone) and local anesthetic (10 mL 0.5% bupivacaine) and varying volumes of saline to achieve full capsular distension or rupture. Despite the existing methodological and procedural differences between the studies, similar to our results, Watson et al. documented the long-term effect of hydrodistention, continuously improving or maintained up to 2 years post-treatment.

After undergoing an initial hydrodistention procedure to treat AC, diabetic patients exhibited a notably higher rate of disease recurrence that necessitated subsequent treatments when compared with those without diabetes. This observed trend resonates with the findings presented by Dimitri-Pinheiro and colleagues. In their study, they documented that a significant proportion, up to 64%, of patients who had diabetes required repeated treatments due to the stability of pain between the repeats after the initial hydrodistention of the glenohumeral joint. In contrast, the percentage was significantly lower for those without diabetes, with only up to 36.3% needing a secondary intervention. Their research, as indicated in reference [6], further underscores the stark difference in treatment recurrence between diabetic and nondiabetic patients after the initial hydrodistention. Nonetheless, this previous work did not evaluate recurrence but rather noneffectiveness of the treatment. Our current results indicate that over the 2 year-evaluation period, patients with diabetes have indeed a higher rate of AC recurrence compared with nondiabetic counterparts.

The pathophysiology of AC in patients with diabetes is still debatable. One hypothesis supports that hyperglycemia may induce changes in the collagen matrix, which have the potential to trigger the fibrotic and inflammatory alterations seen in pathological and histochemical studies of AC [26,27]. Additionally, increased expression of vascular endothelial growth factor and angiogenesis in diabetes-associated AC have also been observed, which may have a role in the pathogenesis and neovascularization of the disease in diabetic patients [28]. Finally, previous reports have suggested an immunological pathogenesis of AC and diabetes mellitus [29]. Although, to our knowledge, the lower time to recurrence of AC in diabetic compared with nondiabetic patients cannot be directly supported by the existing literature, the investigated shared pathogenetic mechanisms between AC and diabetes mellitus may account for the adverse effect of diabetes on the incidence and time of recurrent AC.

Ultrasonography has been found to be valuable for the evaluation of the shoulder to exclude other causes of shoulder pain but not for the diagnosis of AC. Indeed, the diagnosis of AC is not one of the indications for the application of US as indicated by the European Society of Musculoskeletal Radiology guidelines [25]. Certain imaging findings can be seen in patients with AC, such as the reduction of infraspinatus sliding during passive external rotation, thickening of the coracohumeral ligament, increased Doppler flow in the rotator cuff interval, and hypoechoic tissue in the area. These findings are the result of the presence of scar tissue related to the disease. Another finding, usually difficult to demonstrate due to the reduced range of motion of the joint, is the thickening of the axillary recess capsule. The lack of joint effusion is also characteristic in AC [30,31,32]. Despite the fact that US has not been diagnostically established in the management of AC, as demonstrated in our study, it is extremely valuable to guide treatments for AC that achieve long-term patient relief.

US-guided hydrodistention has been performed with either the anterior or the posterior approach. There is evidence that the anterior approach is more effective than the traditional posterior approach. It has been hypothesized that the anterior approach allows the mixture to reach more efficiently the affected areas while also producing better distention and treat biceps tenosynovitis, which many times coexists [33]. In addition, it is technically easier in obese patients and allows direct contact with the patient and evaluation of pain-related facial expressions during the procedure [33]. Moreover, the anterior approach could also help to induce a release of adhesions between the intracapsular portion of the long head of the biceps tendon and the superior (intra-articular) border of the subscapularis tendon.

Our study demonstrated that after US-guided hydrodistention, AC recurred in approximately 8.5% of diabetic compared with 5% of nondiabetic patients, and diabetes was found to be significantly correlated with recurrence. The percentage of recurrence in diabetic patients has been reported to be up to 11% after arthroscopic capsular release, and this has been shown to be higher than nondiabetic counterparts [34,35]. These results are in line with ours, indicating, however, that recurrence following hydrodistention is slightly lower than following arthroscopic release.

Although MR imaging has shown high sensitivity and accuracy in the diagnosis of AC, its high cost, relatively low availability, and the fact that clinical examination combined with US are usually diagnostic, it is not routinely applied before treatment planning (Fields, Zappia). It must be pointed out, though, that many patients at the initial evaluation have already performed an MR imaging study, depending upon the health-care system and its individual diagnostic algorithm.

Our research comes with particular advantages and limitations. The extended 2-year monitoring and its prospective design stand out as significant assets of our investigation. Furthermore, evaluating the recurrence rate of AC within our group presents a fresh insight. Nonetheless, there are definite constraints to note. Not all patients underwent MR imaging study in order to further confirm the diagnosis. One primary limitation is the absence of a no-treatment control group, which would have facilitated a comparison between our participants and the typical progression of AC. Denying the treatment to patients with excruciating pain and reduced range of motion was not considered ethical in our setting. This study is an observational study that could inspire future more controlled studies with a no-treatment control group. In addition, the exclusion of patients with concomitant major shoulder disorders could have affected our results; nonetheless, the inclusion of such patients could be an important confounder with regard to the outcome of hydrodistension. Additionally, the noninclusion of intermediate follow-up time points could be regarded as a limitation, precluding the holistic evaluation of the post-treatment course. However, the short- and midterm effect of hydrodistension has been previously investigated; thus, here we strictly aimed to assess the long-term treatment outcome, which remains under-reported. Another limitation could be the lack of systematic recording of other potential treatments that the patients may have undergone and have not been disclosed, which could affect the outcomes presented here. Finally, future studies with the inclusion of a placebo group would provide insights into the normal course of the disease and the effectiveness of hydrodistension in nondiabetic and diabetic patients in the long term.

## 5. Conclusions

To sum up, our study illuminates the long-term impact of US-guided hydrodistension of the glenohumeral joint. Our results revealed that this therapeutic procedure is efficient in ameliorating shoulder pain while augmenting functional capacity in patients with AC. The longevity of these positive outcomes is noteworthy, as patients continued to experience these benefits for a duration of up to 2 years post-treatment. While these enduring positive effects of the treatment were observed universally across both diabetic and nondiabetic cohorts, a closer examination revealed some pertinent distinctions. Specifically, our analysis suggests that individuals with diabetes, while certainly benefiting from the hydrodistension procedure, displayed a heightened susceptibility to experiencing a recurrence of the disease. Notably, not only was the rate of this recurrence more frequent among diabetic patients, but the interval leading up to this recurrence—the time elapsed since the initial treatment—was also comparatively shorter for them. This contrasts with nondiabetic patients, who seemed to have a more extended period of relief before any potential recurrence of symptoms. Thus, while hydrodistension proves beneficial across the board, the presence of diabetes introduces certain nuances in terms of disease management and expected outcomes.

## Figures and Tables

**Table 1:** Association between the recurrence of adhesive capsulitis and diabetes.

		Diabetes		
		No	Yes	Total
**Recurrence**	**No**	152	22	174
	**Yes**	11	17	28
	**Total**	163	39	202

## Data Availability

Data supporting the reported results are available upon request from the corresponding author of the study.
